# Bis(cyanido-κ*C*)bis­(cyclo­hexyl­amine-κ*N*)mercury(II)

**DOI:** 10.1107/S1600536810001042

**Published:** 2010-01-30

**Authors:** Ejaz, Islam Ullah Khan, William T. A. Harrison

**Affiliations:** a Materials Chemistry Laboratory, Department of Chemistry, GC University, Lahore 54000, Pakistan; b Department of Chemistry, University of Aberdeen, Meston Walk, Aberdeen AB24 3UE, Scotland

## Abstract

In the title compound, [Hg(CN)_2_(C_6_H_13_N)_2_], the Hg^II^ ion adopts an extremely distorted HgC_2_N_2_ tetra­hedral coordination. The crystal packing is influenced by weak N—H⋯N hydrogen bonds between the amino groups and the cyanide N atoms, resulting in chains of mol­ecules propagating in [110]. Both cyclo­hexyl­amine mol­ecules adopt chair conformations.

## Related literature

For related structures, see: Ejaz *et al.* (2009 ▶); Cingolani *et al.* (1987 ▶).
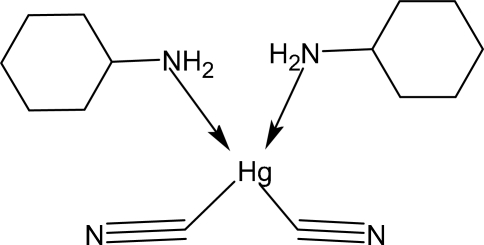


## Experimental

### 

#### Crystal data

[Hg(CN)_2_(C_6_H_13_N)_2_]
*M* *_r_* = 450.98
Triclinic, 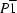

*a* = 7.9283 (4) Å
*b* = 9.1791 (5) Å
*c* = 12.2722 (6) Å
α = 93.972 (3)°
β = 99.179 (3)°
γ = 97.258 (3)°
*V* = 870.95 (8) Å^3^
*Z* = 2
Mo *K*α radiation
μ = 8.83 mm^−1^
*T* = 293 K
0.31 × 0.23 × 0.15 mm

#### Data collection

Bruker Kappa APEXII CCD diffractometer
Absorption correction: multi-scan (*SADABS*; Bruker, 2007 ▶) *T*_min_ = 0.171, *T*_max_ = 0.351
16082 measured reflections
3385 independent reflections
2830 reflections with *I* > 2σ(*I*)
*R*_int_ = 0.041

#### Refinement

*R*[*F* ^2^ > 2σ(*F* ^2^)] = 0.061
*wR*(*F* ^2^) = 0.193
*S* = 1.10
3385 reflections
172 parameters
H-atom parameters constrained
Δρ_max_ = 3.17 e Å^−3^
Δρ_min_ = −1.98 e Å^−3^

### 

Data collection: *APEX2* (Bruker, 2007 ▶); cell refinement: *SAINT* (Bruker, 2007 ▶); data reduction: *SAINT*; program(s) used to solve structure: *SHELXS97* (Sheldrick, 2008 ▶); program(s) used to refine structure: *SHELXL97* (Sheldrick, 2008 ▶); molecular graphics: *ORTEP-3* (Farrugia, 1997 ▶); software used to prepare material for publication: *SHELXL97*.

## Supplementary Material

Crystal structure: contains datablocks I, global. DOI: 10.1107/S1600536810001042/wm2295sup1.cif

Structure factors: contains datablocks I. DOI: 10.1107/S1600536810001042/wm2295Isup2.hkl

Additional supplementary materials:  crystallographic information; 3D view; checkCIF report

## Figures and Tables

**Table:** 

Hg1—C13	2.076 (17)
Hg1—C14	2.084 (17)
Hg1—N2	2.404 (12)
Hg1—N1	2.426 (14)

**Table:** 

C13—Hg1—C14	145.6 (7)
C13—Hg1—N2	100.1 (6)
C14—Hg1—N2	107.0 (7)
C13—Hg1—N1	101.5 (6)
C14—Hg1—N1	102.3 (7)
N2—Hg1—N1	83.4 (5)

**Table 2:** Hydrogen-bond geometry (Å, °)

*D*—H⋯*A*	*D*—H	H⋯*A*	*D*⋯*A*	*D*—H⋯*A*
N1—H2⋯N3^i^	0.90	2.37	3.21 (2)	155
N2—H3⋯N3^i^	0.90	2.48	3.31 (2)	154
N2—H4⋯N4^ii^	0.90	2.37	3.22 (2)	157

Symmetry codes: (i) 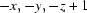; (ii) 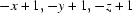.

**Fig. 1.**
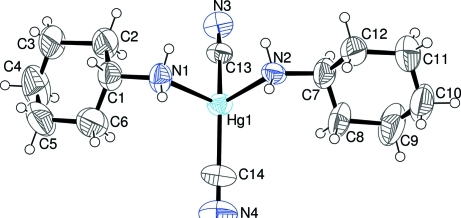
The molecular structure of (I) showing displacement ellipsoids at the 50% probability level.

**Fig. 2.**
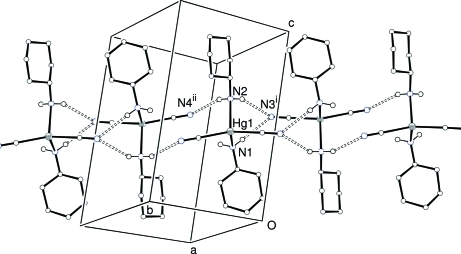
Unit cell packing in (I) with all C-bound hydrogen atoms omitted for clarity and hydrogen bonds indicated by dashed lines. Symmetry codes: (i) –x, –y, 1–z; (ii) 1–x, 1–y, 1–z.

## supplementary crystallographic information

### Comment

As part of our ongoing studies of *MX*_2_Y_2_ complexes (Ejaz *et al.*, 2009), the synthesis and structure of the title compound, (I), (Fig. 1), are now described.

The Hg^II^ atom in (I) adopts what could be described as an extremely distorted HgC_2_N_2_ tetrahedral geometry (Table 1), arising from its coordination by two cyanide anions and two cyclohexylamine ligands. As well as the gross deviations of the bond angles from nominal tetrahedral values, the Hg—C and Hg—N bond lengths are very different. Indeed, an alternative description of the structure of (I) could be to start with a nominal linear Hg(CN)_2_ molecule, which is then weakly coordinated by the two N-bonded ligands (Cingolani *et al.*, 1987). The cyclohexylamine molecules in (I) adopt chair conformations.

In the crystal, the molecules interact by way of N—H···N hydrogen bonds (Table 2), leading to chains in the structure.

Surprisingly, the Cambridge Structural Database contains just one crystal structure containing an Hg(CN)_2_(NR)_2_ unit (Cingolani *et al.*, 1987), in which the C—Hg—C bond angles in the two asymmetric molecules are 148.2 (8) and 163.1 (9)°.

### Experimental

Mercury(II) cyanide (0.5 g, 2.2 mmol) was dissolved in distilled water (20 ml). Cyclohexylamine (0.44 g, 4.4 mmol) was added and the mixture stirred at room temperature for 15 minutes. A white precipitate formed, which was filtered off, washed with distilled water and dried. Colourless blocks of (I) were recrystallized from methanol.

### Refinement

All the hydrogen atoms were placed in calculated positions (C—H = 0.97–0.98 Å, N—H = 0.90 Å) and refined as riding with *U*_iso_(H) = 1.2*U*_eq_(carrier). The highest difference peak is 1.54Å from N3 and the deepest difference hole is 0.89Å from H1A.

### Crystal data

**Table:**

[Hg(CN)_2_(C_6_H_13_N)_2_]	*V* = 870.95 (8) Å^3^
*M**_r_* = 450.98	*Z* = 2
Triclinic, *P*1	*F*(000) = 436
Hall symbol: -P 1	*D*_x_ = 1.720 Mg m^−^^3^
*a* = 7.9283 (4) Å	Mo *K*α radiation, λ = 0.71073 Å
*b* = 9.1791 (5) Å	µ = 8.83 mm^−^^1^
*c* = 12.2722 (6) Å	*T* = 293 K
α = 93.972 (3)°	Block, colourless
β = 99.179 (3)°	0.31 × 0.23 × 0.15 mm
γ = 97.258 (3)°	

### Data collection

**Table:**

Bruker Kappa APEXII CCD diffractometer	3385 independent reflections
Radiation source: fine-focus sealed tube	2830 reflections with *I* > 2σ(*I*)
graphite	*R*_int_ = 0.041
ω scans	θ_max_ = 26.0°, θ_min_ = 2.6°
Absorption correction: multi-scan (*SADABS*; Bruker, 2007)	*h* = −9→9
*T*_min_ = 0.171, *T*_max_ = 0.351	*k* = −11→11
16082 measured reflections	*l* = −15→15

### Refinement

**Table:**

Refinement on *F*^2^	Primary atom site location: structure-invariant direct methods
Least-squares matrix: full	Secondary atom site location: difference Fourier map
*R*[*F*^2^ > 2σ(*F*^2^)] = 0.061	Hydrogen site location: inferred from neighbouring sites
*wR*(*F*^2^) = 0.193	H-atom parameters constrained
*S* = 1.10	*w* = 1/[σ^2^(*F*_o_^2^) + (0.0858*P*)^2^ + 12.3401*P*] where *P* = (*F*_o_^2^ + 2*F*_c_^2^)/3
3385 reflections	(Δ/σ)_max_ = 0.001
172 parameters	Δρ_max_ = 3.17 e Å^−^^3^
0 restraints	Δρ_min_ = −1.98 e Å^−^^3^

### Special details

**Table:**

Geometry. All e.s.d.'s (except the e.s.d. in the dihedral angle between two l.s. planes) are estimated using the full covariance matrix. The cell e.s.d.'s are taken into account individually in the estimation of e.s.d.'s in distances, angles and torsion angles; correlations between e.s.d.'s in cell parameters are only used when they are defined by crystal symmetry. An approximate (isotropic) treatment of cell e.s.d.'s is used for estimating e.s.d.'s involving l.s. planes.
Refinement. Refinement of *F*^2^ against ALL reflections. The weighted *R*-factor *wR* and goodness of fit *S* are based on *F*^2^, conventional *R*-factors *R* are based on *F*, with *F* set to zero for negative *F*^2^. The threshold expression of *F*^2^ > σ(*F*^2^) is used only for calculating *R*-factors(gt) *etc*. and is not relevant to the choice of reflections for refinement. *R*-factors based on *F*^2^ are statistically about twice as large as those based on *F*, and *R*- factors based on ALL data will be even larger.

### Fractional atomic coordinates and isotropic or equivalent isotropic displacement parameters (Å^2^)

**Table:**

	*x*	*y*	*z*	*U*_iso_*/*U*_eq_	
Hg1	0.15752 (8)	0.30488 (6)	0.45191 (5)	0.0641 (3)	
C1	0.363 (3)	0.059 (2)	0.3106 (15)	0.082 (5)	
H1A	0.4532	−0.0049	0.3212	0.099*	
C2	0.199 (4)	−0.038 (3)	0.272 (2)	0.132 (11)	
H2A	0.1036	0.0184	0.2616	0.159*	
H2B	0.1784	−0.1096	0.3247	0.159*	
C3	0.221 (5)	−0.119 (3)	0.158 (2)	0.155 (14)	
H3A	0.3180	−0.1736	0.1694	0.187*	
H3B	0.1180	−0.1884	0.1289	0.187*	
C4	0.249 (4)	−0.013 (4)	0.077 (2)	0.148 (12)	
H4A	0.1556	0.0462	0.0670	0.177*	
H4B	0.2538	−0.0644	0.0057	0.177*	
C5	0.420 (6)	0.083 (4)	0.123 (3)	0.176 (17)	
H5A	0.4461	0.1538	0.0709	0.211*	
H5B	0.5116	0.0215	0.1318	0.211*	
C6	0.414 (4)	0.166 (3)	0.236 (2)	0.125 (9)	
H6A	0.5268	0.2198	0.2672	0.150*	
H6B	0.3320	0.2358	0.2269	0.150*	
N1	0.3599 (18)	0.1352 (17)	0.4211 (11)	0.074 (4)	
H1	0.4662	0.1852	0.4446	0.089*	
H2	0.3457	0.0643	0.4673	0.089*	
C7	0.197 (3)	0.355 (2)	0.7345 (14)	0.085 (5)	
H7	0.0734	0.3208	0.7271	0.102*	
C8	0.220 (3)	0.509 (2)	0.7259 (16)	0.096 (6)	
H8A	0.1653	0.5275	0.6528	0.115*	
H8B	0.3425	0.5431	0.7331	0.115*	
C9	0.145 (5)	0.598 (3)	0.814 (2)	0.150 (13)	
H9A	0.1665	0.7025	0.8056	0.180*	
H9B	0.0215	0.5686	0.8061	0.180*	
C10	0.232 (5)	0.566 (3)	0.925 (2)	0.134 (10)	
H10A	0.3523	0.6092	0.9350	0.160*	
H10B	0.1798	0.6142	0.9812	0.160*	
C11	0.222 (5)	0.407 (3)	0.940 (2)	0.137 (11)	
H11A	0.1041	0.3673	0.9433	0.164*	
H11B	0.2931	0.3946	1.0101	0.164*	
C12	0.284 (3)	0.324 (2)	0.8463 (15)	0.097 (6)	
H12A	0.4076	0.3512	0.8523	0.117*	
H12B	0.2619	0.2191	0.8534	0.117*	
N2	0.2619 (18)	0.2820 (14)	0.6440 (9)	0.068 (3)	
H3	0.2471	0.1849	0.6529	0.082*	
H4	0.3766	0.3111	0.6552	0.082*	
C13	−0.073 (2)	0.1629 (18)	0.4292 (15)	0.067 (4)	
N3	−0.196 (2)	0.0797 (19)	0.4154 (15)	0.090 (4)	
C14	0.282 (2)	0.504 (2)	0.4133 (18)	0.089 (6)	
N4	0.352 (3)	0.610 (2)	0.395 (2)	0.116 (6)	

### Atomic displacement parameters (Å^2^)

**Table:**

	*U*^11^	*U*^22^	*U*^33^	*U*^12^	*U*^13^	*U*^23^
Hg1	0.0633 (4)	0.0592 (4)	0.0654 (4)	−0.0021 (2)	0.0064 (3)	0.0050 (3)
C1	0.080 (11)	0.096 (13)	0.067 (10)	0.004 (10)	0.014 (9)	−0.006 (9)
C2	0.16 (2)	0.111 (17)	0.118 (19)	−0.051 (16)	0.064 (18)	−0.043 (15)
C3	0.24 (4)	0.117 (19)	0.090 (17)	−0.06 (2)	0.06 (2)	−0.061 (16)
C4	0.15 (2)	0.22 (4)	0.058 (13)	0.00 (2)	0.005 (14)	−0.031 (18)
C5	0.26 (5)	0.16 (3)	0.11 (2)	−0.05 (3)	0.11 (3)	−0.016 (19)
C6	0.16 (2)	0.118 (19)	0.086 (15)	−0.035 (17)	0.026 (15)	−0.003 (13)
N1	0.069 (8)	0.088 (9)	0.060 (8)	0.005 (7)	0.009 (6)	−0.014 (7)
C7	0.111 (15)	0.088 (12)	0.052 (9)	0.017 (11)	0.007 (9)	−0.010 (8)
C8	0.121 (16)	0.095 (14)	0.065 (11)	0.037 (12)	−0.005 (10)	−0.023 (10)
C9	0.21 (3)	0.13 (2)	0.11 (2)	0.09 (2)	0.02 (2)	−0.026 (17)
C10	0.20 (3)	0.12 (2)	0.083 (16)	0.03 (2)	0.025 (18)	−0.032 (14)
C11	0.20 (3)	0.13 (2)	0.074 (14)	−0.01 (2)	0.041 (17)	−0.019 (14)
C12	0.142 (19)	0.086 (13)	0.061 (11)	0.013 (13)	0.013 (11)	0.003 (9)
N2	0.086 (9)	0.064 (7)	0.045 (6)	−0.024 (6)	0.008 (6)	0.005 (5)
C13	0.056 (9)	0.064 (9)	0.083 (11)	0.010 (7)	0.015 (8)	0.018 (8)
N3	0.072 (10)	0.090 (11)	0.103 (12)	0.013 (9)	0.001 (8)	0.012 (9)
C14	0.067 (10)	0.084 (12)	0.114 (15)	−0.004 (9)	0.007 (10)	0.048 (11)
N4	0.090 (12)	0.100 (13)	0.153 (19)	−0.005 (10)	0.013 (12)	0.034 (13)

### Geometric parameters (Å, °)

**Table:**

Hg1—C13	2.076 (17)	N1—H2	0.9000
Hg1—C14	2.084 (17)	C7—C8	1.41 (3)
Hg1—N2	2.404 (12)	C7—N2	1.45 (2)
Hg1—N1	2.426 (14)	C7—C12	1.50 (3)
C1—C6	1.45 (3)	C7—H7	0.9800
C1—C2	1.47 (3)	C8—C9	1.55 (3)
C1—N1	1.49 (2)	C8—H8A	0.9700
C1—H1A	0.9800	C8—H8B	0.9700
C2—C3	1.58 (3)	C9—C10	1.48 (4)
C2—H2A	0.9700	C9—H9A	0.9700
C2—H2B	0.9700	C9—H9B	0.9700
C3—C4	1.45 (4)	C10—C11	1.48 (4)
C3—H3A	0.9700	C10—H10A	0.9700
C3—H3B	0.9700	C10—H10B	0.9700
C4—C5	1.53 (4)	C11—C12	1.52 (3)
C4—H4A	0.9700	C11—H11A	0.9700
C4—H4B	0.9700	C11—H11B	0.9700
C5—C6	1.55 (3)	C12—H12A	0.9700
C5—H5A	0.9700	C12—H12B	0.9700
C5—H5B	0.9700	N2—H3	0.9000
C6—H6A	0.9700	N2—H4	0.9000
C6—H6B	0.9700	C13—N3	1.14 (2)
N1—H1	0.9000	C14—N4	1.12 (2)
			
C13—Hg1—C14	145.6 (7)	Hg1—N1—H2	106.6
C13—Hg1—N2	100.1 (6)	H1—N1—H2	106.5
C14—Hg1—N2	107.0 (7)	C8—C7—N2	109.0 (17)
C13—Hg1—N1	101.5 (6)	C8—C7—C12	109.4 (16)
C14—Hg1—N1	102.3 (7)	N2—C7—C12	113.0 (17)
N2—Hg1—N1	83.4 (5)	C8—C7—H7	108.4
C6—C1—C2	116 (2)	N2—C7—H7	108.4
C6—C1—N1	109.7 (17)	C12—C7—H7	108.4
C2—C1—N1	109.9 (16)	C7—C8—C9	114 (2)
C6—C1—H1A	106.8	C7—C8—H8A	108.8
C2—C1—H1A	106.8	C9—C8—H8A	108.8
N1—C1—H1A	106.8	C7—C8—H8B	108.8
C1—C2—C3	105 (2)	C9—C8—H8B	108.8
C1—C2—H2A	110.7	H8A—C8—H8B	107.7
C3—C2—H2A	110.7	C10—C9—C8	107 (2)
C1—C2—H2B	110.7	C10—C9—H9A	110.3
C3—C2—H2B	110.7	C8—C9—H9A	110.3
H2A—C2—H2B	108.8	C10—C9—H9B	110.3
C4—C3—C2	111 (2)	C8—C9—H9B	110.3
C4—C3—H3A	109.4	H9A—C9—H9B	108.5
C2—C3—H3A	109.4	C11—C10—C9	114 (2)
C4—C3—H3B	109.4	C11—C10—H10A	108.8
C2—C3—H3B	109.4	C9—C10—H10A	108.8
H3A—C3—H3B	108.0	C11—C10—H10B	108.8
C3—C4—C5	106 (3)	C9—C10—H10B	108.8
C3—C4—H4A	110.5	H10A—C10—H10B	107.7
C5—C4—H4A	110.5	C10—C11—C12	111 (2)
C3—C4—H4B	110.5	C10—C11—H11A	109.4
C5—C4—H4B	110.5	C12—C11—H11A	109.4
H4A—C4—H4B	108.7	C10—C11—H11B	109.4
C4—C5—C6	111 (3)	C12—C11—H11B	109.4
C4—C5—H5A	109.4	H11A—C11—H11B	108.0
C6—C5—H5A	109.4	C7—C12—C11	113 (2)
C4—C5—H5B	109.4	C7—C12—H12A	109.1
C6—C5—H5B	109.4	C11—C12—H12A	109.1
H5A—C5—H5B	108.0	C7—C12—H12B	109.1
C1—C6—C5	109 (2)	C11—C12—H12B	109.1
C1—C6—H6A	110.0	H12A—C12—H12B	107.8
C5—C6—H6A	110.0	C7—N2—Hg1	123.3 (12)
C1—C6—H6B	110.0	C7—N2—H3	106.5
C5—C6—H6B	110.0	Hg1—N2—H3	106.5
H6A—C6—H6B	108.4	C7—N2—H4	106.5
C1—N1—Hg1	123.1 (12)	Hg1—N2—H4	106.5
C1—N1—H1	106.6	H3—N2—H4	106.5
Hg1—N1—H1	106.6	N3—C13—Hg1	177.0 (15)
C1—N1—H2	106.6	N4—C14—Hg1	178.2 (19)
			
C6—C1—C2—C3	57 (3)	N2—C7—C8—C9	176 (2)
N1—C1—C2—C3	−177 (2)	C12—C7—C8—C9	−60 (3)
C1—C2—C3—C4	−62 (4)	C7—C8—C9—C10	59 (3)
C2—C3—C4—C5	64 (4)	C8—C9—C10—C11	−53 (4)
C3—C4—C5—C6	−60 (4)	C9—C10—C11—C12	52 (4)
C2—C1—C6—C5	−56 (4)	C8—C7—C12—C11	55 (3)
N1—C1—C6—C5	179 (3)	N2—C7—C12—C11	177 (2)
C4—C5—C6—C1	55 (4)	C10—C11—C12—C7	−51 (3)
C6—C1—N1—Hg1	66 (2)	C8—C7—N2—Hg1	−58 (2)
C2—C1—N1—Hg1	−63 (2)	C12—C7—N2—Hg1	−179.6 (13)
C13—Hg1—N1—C1	69.3 (14)	C13—Hg1—N2—C7	−77.6 (14)
C14—Hg1—N1—C1	−85.7 (14)	C14—Hg1—N2—C7	80.9 (14)
N2—Hg1—N1—C1	168.3 (14)	N1—Hg1—N2—C7	−178.2 (14)

### Hydrogen-bond geometry (Å, °)

**Table:**

*D*—H···*A*	*D*—H	H···*A*	*D*···*A*	*D*—H···*A*
N1—H2···N3^i^	0.90	2.37	3.21 (2)	155
N2—H3···N3^i^	0.90	2.48	3.31 (2)	154
N2—H4···N4^ii^	0.90	2.37	3.22 (2)	157

Symmetry codes: (i) −*x*, −*y*, −*z*+1; (ii) −*x*+1, −*y*+1, −*z*+1.

## References

[bb1] Bruker (2007). *APEX2*, *SAINT* and *SADABS* Bruker AXS Inc., Madison, Wisconsin, USA.

[bb2] Cingolani, A., Lorenzotti, A., Lobbia, G. G., Leonesi, D., Bonati, F. & Bovio, B. (1987). *Inorg. Chim. Acta*, **132**, 167–176.

[bb3] Ejaz, Sahin, O. & Khan, I. U. (2009). *Acta Cryst.* E**65**, m1457. 10.1107/S1600536809043694 PMC2971042 21578185

[bb4] Farrugia, L. J. (1997). *J. Appl. Cryst.* **30**, 565.

[bb5] Sheldrick, G. M. (2008). *Acta Cryst.* A**64**, 112–122. 10.1107/S0108767307043930 18156677

